# Detection of Antibodies for Pathogenic *Leptospira* in Wild Mammals and Birds from Southern Chile—First Record of Seropositivity in a Guiña (*Leopardus guigna*)

**DOI:** 10.3390/ani14040601

**Published:** 2024-02-12

**Authors:** Luis Balcázar, Lucía Azócar-Aedo, Violeta Barrera, Gloria Meniconi, Victoria Muñoz, Carola Valencia-Soto

**Affiliations:** 1 Facultad de Ciencias de la Naturaleza, Universidad San Sebastián Sede de la Patagonia, Puerto Montt 5480000, Chile; 2 Parque Nacional Puyehue, Corporación Nacional Forestal (CONAF), Ruta 215, Puyehue 5360000, Chile

**Keywords:** *Leptospira* spp., zoonosis, wildlife, birds, mammals, seroprevalence, Chile

## Abstract

**Simple Summary:**

Wild animals can harbor infectious agents for several diseases. Some of these pathologies are zoonotic because they can be transmitted from animals to humans. Among these, leptospirosis is a zoonosis transmitted by bacteria of the genus *Leptospira*, which is present worldwide. In this study, animals at a wildlife rehabilitation center in Puerto Montt, southern Chile, were sampled from January to May 2023. Sixty animals, including birds, mammals, and reptiles, were selected to determine the prevalence of pathogenic *Leptospira* using a diagnostic test that detects antibodies for the bacteria. The prevalence was 25.00% in the total number of animals sampled, with prevalence of 19.51% in birds (choroy parrots, bandurrias, and Magellanic penguins) and a frequency of 38.89% in mammals (chilla foxes, pudus, and guiñas). Seropositivity to the bacteria is reported for the first time in guiñas. These results indicate that different wild animal species are exposed to *Leptospira* and that increased awareness about the disease is needed. Prevention measures must be implemented to reduce the risk of transmission between animals, as well as from animals to humans.

**Abstract:**

Leptospirosis is a neglected bacterial zoonotic disease of worldwide distribution that is present in different animal species. This epidemiological study determined the seroprevalence of pathogenic *Leptospira* spp. in animals at a wildlife rehabilitation center in Puerto Montt, southern Chile, by sampling 60 animals belonging to three classes (birds, mammals, and reptiles). Diagnosis was performed using the microscopic agglutination test with a panel of eight serovars and serogroups. The results showed that 15 animals had anti-*Leptospira* antibodies, obtaining a seroprevalence of 25.00%, with *Leptospira borgpetersenii* serogroup Tarassovi presenting reactivity in 13 of the seropositive animals. Among the classes of mammals, chilla foxes (*Lycalopex griseus*) and pudus (*Pudu puda*) were seropositive. A guiña (*Leopardus guigna*) was also seropositive, which was described for the first time in mammals. Among the classes of birds, choroy parrots (*Enicognathus leptorhynchus*), bandurrias (*Theristicus melanopis*), and Magellanic penguins *(Spheniscus magellanicus*) were seropositive. Routine examinations to diagnose leptospirosis, perform epidemiological surveillance, and apply prevention and control measures are necessary, and additional research focusing on the One Health approach to explore the epidemiological role of different wild animal species in the maintenance and transmission of leptospirosis at the local and global levels are recommended.

## 1. Introduction

Leptospirosis is a zoonotic disease caused by bacteria of the genus *Leptospira* [1], which is divided into three subgroups, namely, pathogenic, saprophytic, and intermediate species, which are those with unclear pathogenicity [2]. Currently, the genus *Leptospira* comprises more than 300 serovars organized into 30 serogroups [1].

Leptospires have been detected in different animals, including mammals, reptiles, amphibians, fishes, and invertebrates [3]. The animal species that are considered important sources of infection are small mammals, especially wild and synanthropic rodents (rats and mice), followed by insectivorous mammals (shrews and hedgehogs) and domestic animals (cattle, pigs, canines, sheep, goats, and equines) [4], with the Norwegian rat mouse (*Rattus norvegicus*) being the largest source of human infections [5]. Leptospires reside in the renal tubules of the hosts, and they horizontally transmit the bacteria to susceptible animals that become infected directly or indirectly through contact with urine or the environment being contaminated by urine [4].

Leptospirosis is a major public health problem that occurs at the animal–human–environment ecosystem interface, and its epidemiology is complex because of geographic and host factors [6]. Wild animals can participate in the epidemiological chain of leptospirosis as reservoirs, maintenance hosts, and incidental hosts, depending on the characteristics specific to the species, environmental conditions, and susceptibility of animals to infection [7]. Serogroups and serovars found in wildlife are usually associated with domestic animals that are reservoirs in the area, such as cattle, pigs, and dogs [8].

Southern Chile has properties that allow the presentation and permanence of leptospires in the hosts and environment, including the presence of wild animals that are potential reservoirs, the existence of domestic animals that are in contact with wild fauna, and the abundance of wetlands and watercourses [9]. The first study of leptospirosis in wild animals in Chile was conducted in 1938, where negative results were obtained, presumably because of the use of only one serovar as an antigen in the microscopic agglutination test (MAT). In the 1950s, studies estimated the prevalence of *Leptospira* in rodents and foxes. In the 1990s, a survey was conducted on 784 wild rodents in the city of Valdivia (southern Chile) for three years, obtaining a prevalence of 37.8%, which varied according to the season of the year when the rodents were captured, increasing in the seasons with greater rainfall levels with frequencies of 41.4%, 37.3%, and 29.8% in winter, spring and autumn, and summer, respectively [10].

Currently, in Chile, rodents continue to predominate in epidemiological studies of wildlife, with evidence of infection in six native species, namely, the degu (*Octodon degus*), olive mouse (*Abrothrix olivaceus*), fur long-tailed mouse (*Abrothrix longipilis*), long-eared mouse (*Phyllotis darwini*), long-tailed mouse (*Oligoryzomys longicaudatus*), and Valdivian mole mouse (*Geoxus valdivianus*), and in three introduced species, namely, the black rat (*Rattus rattus*), Norwegian rat (*Rattus norvegicus*), and house mouse (*Mus musculus*). In other orders of mammals, seropositivity has been reported in six native species, namely, the South American sea lion (*Otaria flavescens*), guanaco (*Lama guanicoe*), pudu (*Pudu puda*), culpeo fox (*Lycalopex culpaeus*), chilla fox (*Lycalopex griseus*), and puma (*Puma concolor*), and in an introduced species, that is, the American mink (*Neovison vison*) [10,11,12,13].

The main reservoirs of *Leptospira* spp., the most common serovars and strains harbored by wildlife, and their relationship to human diseases are still unknown [14]. Considering that the identification of the leptospiral serovars and serogroups involved in the infection provides clues regarding the reservoirs that played a role in the transmission of the bacteria [2] and that more research is necessary in wild animals to provide information on the maintenance and transmission of *Leptospira* spp. to understand the dynamics of the pathogen [12], the aims of this study were to (1) determine the seroprevalence of pathogenic *Leptospira* in wild animals at a wildlife rehabilitation center in southern Chile using the MAT, (2) evaluate the serological reactivity and antibody titers for eight pathogenic *Leptospira* serogroups using the MAT, and (3) identify demographic characteristics related to seropositive animals.

## 2. Materials and Methods

### 2.1. Type of Study

This research was a cross-sectional, descriptive, and quantitative observational epidemiological study [15].

### 2.2. Study Area

Samples were collected at a wildlife rehabilitation center of the Universidad San Sebastián (USS), campus Patagonia, in the city of Puerto Montt, Los Lagos Region, southern Chile. Wild animals were brought to this center from various cities and places in the region, derived and transported by the Servicio Agrícola y Ganadero and the Servicio Nacional de Pesca y Acuicultura of Chile.

### 2.3. Population and Sample

The study population consisted of different classes (birds, mammals, and reptiles) and species of wild animals that were in the wildlife rehabilitation center during the sampling period from January to May 2023.

Sample size was estimated using WinEpi [16], considering that each year, 200 animals enter the wildlife rehabilitation center. With expected prevalence of 50.0%, a confidence level of 95%, and an accepted error of 11.0%, the calculated sample size was 57 animals. Finally, 60 individuals were sampled.

### 2.4. Inclusion and Exclusion Criteria

As an inclusion criterion, animals with a physiological and clinical status compatible with the holding and extraction of blood samples were considered suitable for the study.

As exclusion criteria, patients who presented with phlebitis in the veins of choice for sample collection and/or severe dehydration were considered unsuitable for this study. Animals in which 1% of their live weight was <1 g were also excluded.

### 2.5. Sample Collection

Blood samples from the peripheral veins of the animals (0.5–1 mL) were obtained and stored in numbered tubes without an anticoagulant at 4 °C. The species, tube number, and demographic data of the animals were recorded (age, place of origin, and reason for the admission to the wildlife rehabilitation center). Subsequently, the samples were centrifuged for 5 min at 3000 rpm to separate the serum from other blood components and then were stored at −20 °C until analysis.

### 2.6. MAT

The MAT was conducted for the diagnosis of seropositivity on pathogenic *Leptospira* spp. following a standard protocol established by Faine, the World Health Organization, and the International Leptospirosis Society (ILS) [4,17]. A sample was classified as positive if it showed 50% agglutination. Then, the titer of anti-*Leptospira* antibodies for each serovar was determined using the positive samples, considering 1:100, 1:200, 1:400, 1:800, and 1:1600 dilutions.

A panel of eight serogroups and serovars of pathogenic *Leptospira* were used as antigens: *Leptospira interrogans* serovars Hardjo (serogroup Sejroe), Pomona (serogroup Pomona), Canicola (serogroup Canicola), Icterohaemorrhagiae (serogroup Icterohaemorrhagiae), Autumnalis (serogroup Autumnalis), and Bratislava (serogroup Australis); *Leptospira borgpetersenii* serovar Tarassovi (serogroup Tarassovi); and *Leptospira kirschneri* serovar Grippotyphosa (serogroup Grippotyphosa).

### 2.7. Interpreting the MAT Results

Titers from 1:100 were considered seropositive for pathogenic Leptospira. If a sample reacted to more than one serogroup, the serogroup with the highest titer was considered the cause of seropositivity, and if a sample presented the same antibody titer for two different serogroups, it was classified as coagglutination [1,4].

### 2.8. Data Analysis

The seroprevalence of the sampled animals was estimated using the following modified formula [18]:

Prevalence = (Number of positive animals in a period/Total number of individuals sampled in the same period) × Factor.

Furthermore, 95% confidence intervals (95% CI) were calculated for seroprevalence following the methodology of Noordiuzen et al. [19].

Possible differences in seroprevalence values according to the class and species of the animals, *Leptospira* serogroups, antibody titers, and demographic characteristics of the seropositive individuals were determined using the chi-square test with the Yates correction [20], considering *p* values < 0.05 as statistically significant. All statistical analyses were performed using Epi Info version 6.04.

## 3. Results

### 3.1. Seropoprevalence of Pathogenic Leptospira in Wild Animals Using the MAT

The samples included three different classes of wild animals: 12 bird species, 5 mammal species, and 1 reptile species.

The samples were obtained from 41 individuals of the bird group, 18 corresponding to the mammal group, and 1 representative of reptiles, with a total of 60 animals studied.

Anti-*Leptospira* antibodies were found in 15 animals that belong to two of the three classes sampled, resulting in overall seroprevalence of 25.00% (95% CI = 14.04–35.96). The seroprevalence values in birds, mammals, and reptiles were 19.51% (95% CI = 7.37–31.64), 38.89% (95% CI = 16.37–61.40), and 0%, respectively (Table 1). No statistically significant differences in the seroprevalence values between classes (*p* value > 0.05) were found. Of the 15 positive animals, 8 (53.33%) were birds and 7 (46.67%) were mammals.

In particular, three bird species showed serological reactivity to pathogenic *Leptospira* spp.: choroy parrot (*Enicognathus leptorhynchus*), bandurria (*Theristicus melanopis*), and Magellanic penguin (*Spheniscus magellanicus*), with seroprevalence values of 33.33% (95% CI = 2.53–64.13), 23.08% (95% CI = 0.18–45.98), and 66.67% (95% CI = 13.33–100), respectively (Table 2). These differences were not statistically significant (*p* > 0.05).

In mammals, three species also showed reactivity to *Leptospira*: chilla fox (*Lycalopex griseus*), 44.44% (95% CI = 11.98–76.90); pudu (*Pudu puda*), 40.00% (95% CI = 0.0–82.94); and guiña (*Leopardus guigna*), 50.00% (95% CI = 0.0–100) (Table 2). The differences were not statistically significant (*p* > 0.05).

### 3.2. Serological Reactivity and Antibody Titers of the Serogroups of Pathogenic Leptospira

Of the 15 seropositive animals, 14 had antibody titers for more than 1 serogroup, indicating cross-infections. The number of samples reactive to each of the serogroups used in the MAT from the highest to the lowest was 12 (80.00%) for Tarassovi, 1 (6.67%) for Grippotyphosa, 1 (6.67%) for Sejroe, and 1 (6.67%) for coagglutination between Tarassovi and Sejroe. The differences between the mentioned proportions were not statistically significant (*p* > 0.05). No serological reactions were observed for the serogroups Pomona, Canicola, Icterohaemorrhagiae, Autumnalis, and Australis.

The lowest and highest antibody titers were 1:100 and 1:1600, respectively. The minimum reactivity was detected in the sample of a bandurria against the serogroup Grippotyphosa. Conversely, the highest antibody titer was obtained from the sample of a Magellanic penguin against the serogroup Tarassovi (Table 3). The frequencies of presentation of seropositivity for each antibody level were the following: 1:100 (6.67%), 1:200 (13.33%), 1:400 (46.67%), 1:800 (26.67%), and 1:1600 (6.67%). No statistically significant differences were found between these proportions (*p* value > 0.05).

### 3.3. Demographic Characteristics of Animals Seropositive for Pathogenic Leptospira

Demographic characteristics included the species, place of origin, age, and reason for the admission of the animals to the wildlife rehabilitation center. The presentation of sex as an antecedent was ruled out because of the difficulty of determining it, especially in birds without sexual dimorphism.

All of the cities of origin of the seropositive animals belonged to the Los Lagos region, where Osorno city was the most common origin (40.00%).

The most frequent age group among seropositive animals was adults (66.67%). The youth age group accounted for 33.33% of the seropositive animals.

The reasons for the admission to the wildlife rehabilitation center were diverse, including some related to multiple traumas and injuries, for example, prostration (26.67%), being hit by a car (13.33%), attacked by dogs (13.33%), animals found stranded (13.33%), as well as other issues such as unconsciousness, lethargy, wing injury, and orphanhood (6.67% each one).

## 4. Discussion

In this study, wild animals at the wildlife rehabilitation center of the USS, campus Patagonia, Puerto Montt, in southern Chile, were sampled. Sixty animals, including birds, mammals, and reptiles, were selected to determine the seroprevalence of pathogenic *Leptospira* and antibody titers for eight serogroups using the MAT. Seropositivity was then compared between classes and species, and some demographic characteristics related to seropositive animals were identified. The overall seroprevalence was 25.00% in the sampled animals, with 19.51%, 38.89%, and 0% seroprevalence in birds, mammals, and reptiles, respectively. Seropositivity in different species was found, including choroy parrots, bandurrias, Magellanic penguins, chilla foxes, and pudus, and the presence of anti-*Leptospira* antibodies in a guiña was described for the first time. The seroprevalence found is close to that of a study in Slovenia, in which an analysis of 249 blood sera from wild animals revealed antibodies to at least one of the pathogenic serovars in 30.9% of the samples [21].

A study in a zoo in the metropolitan region in Chile that involved exotic and native mammals determined *Leptospira* seropositivity in three of the seven pudus sampled (42.86%) with titers for the serovars Grippotyphosa, Bratislava, and Autumnalis, with Autumnalis being the most frequent [13]. In the present investigation, seropositivity was determined in two of the five pudus sampled (40.00%) and the most frequent serogroup responsible for the serological reactions was Tarassovi. According to the IUCN Red List of Threatened Species [22], *Pudu puda* is listed as a “near threatened species”. In these animals, the association between *Leptospira* infection and clinical diseases, the clinical manifestations that will develop, and the antibody titers that are consistent with an active infection are unknown, which is relevant to investigate in future research to elucidate the importance of leptospirosis as a cause of morbidity or even mortality in these animals.

In the 1950s, seroprevalence of 7.4% was found in culpeo and chilla foxes for the serovars Grippotyphosa, Icterohaemorrhagiae, and Copenhageni in Chile [10]. In another study conducted in foxes from Tierra del Fuego, anti-*Leptospira* antibodies were detected in 20.0% and 8.0% of the culpeo and chilla foxes sampled, respectively [23]. In a more recent study, the prevalence of *Leptospira* spp. from 46 samples of culpeo and chilla foxes was 7.7% [24]. In our research, seroprevalence of 44.44% was found, which represents the highest frequency for *Leptospira* reported so far in Chile for chilla foxes. Of the four seropositive animals, two were reactive for the serogroup Tarassovi, with titers of 1:400. In addition, one animal was positive for the serogroup Sejroe with a titer of 1:800, and there was coagglutination between Tarassovi and Sejroe with a titer of 1:400. The greater seropositivity to the serogroup Tarassovi could be associated with the contact of the animals with pigs or wild boars, which are described as the maintenance host for the serovar [25]. Transmission pathways differ among animal species, as well as across rural and urban landscapes and environmental conditions, which influence the survival of pathogenic *Leptospira* [26]. Southern Chile presents ecological and climatic conditions that favor the presence of leptospires in the environment, such as high rainfall and humidity [9], which could explain the seroprevalence found. Another reason could be the exposure of chilla foxes to animals such as domestic animals in the wildlife habitat, which is common in rural areas in southern Chile. The modification of the ecosystems and wildlife habitats and the constantly increasing number of animal species moving toward urban or peri-urban areas increase the possibility of direct or indirect contacts between wildlife and domestic animals [25].

According to the literature, this is the first study that reported seropositivity to pathogenic *Leptospira* in the guiña species (*Leopardus guigna*) that had seroreactivity to the serogroup Tarassovi. In a relatively recent investigation conducted in two wildlife holding centers in Chile, namely, one in the Los Ríos region and the other in the Los Lagos region, 11 pumas and 3 guiñas were sampled, resulting in seropositivity in only 4 pumas against the serogroup Autumnalis [11]. More epidemiological studies are needed to characterize the presentation of *Leptospira* infection in guiñas. *Leopardus guigna* has been listed on the IUCN Red List of Threatened Species as “vulnerable” under criteria A2abc; C2a(i) [22]. It is well established that the threat of a disease increases as a species becomes more endangered, but this is unknown for leptospirosis [27]. Therefore, it is important to determine the relevance of leptospirosis as a risk factor for an increase in morbidity and to elucidate the health consequences of the infection. The seropositive animal found in this study presented antibodies against the serogroup Tarassovi, with titers of 1:800. This antibody level is associated with a confirmatory diagnosis of leptospirosis from a single serum sample in species such as domestic dogs (*Canis lupus familiaris*) [28].

In this study, the South American sea lion was found to have no anti-*Leptospira* antibodies. By contrast, through immunohistochemistry in the research conducted in Valdivia, Chile, a prevalence of 33.33% (1/3) was detected in sea lions [29].

Research on leptospirosis in birds is scarce, and reports of seropositivity in epidemiological studies are uncommon. The importance of birds in the epidemiology of *Leptospira* infection and the clinical manifestations of the disease and their role in transmission to other birds or animals are unknown. One description of the infection in birds was published by Davis [30] and Ormsbee et al. [31], who noted that embryonated eggs from domestic chickens can be infected by chorioallantoic inoculation from days 9–12, and petechial hemorrhages can be observed within 48–72 h post *Leptospira* infection. In a more updated systematic review on leptospirosis in studies conducted in Latin America, it was found that a small number of birds were studied in two published articles, in which 12 animals were sampled and all of them were seronegative [14]. Conversely, in a survey in Botswana, *Leptospira* spp. were found in kidney samples from 5 of the 18 bird species analyzed (27.8%) [32]. We found anti-*Leptospira* antibody titers in choroy parrots (*Enicognathus leptorhynchus*), bandurrias (*Theristicus melanopis*), and Magellanic penguins (*Spheniscus magellanicus*). In a study conducted on Magdalena Island, Chile, 132 serum samples were collected from Magellanic penguins and none was seropositive [33]. The possible differences in the seroprevalence values could be due to the MAT panel used in both studies, the cutoff of the antibody titers considered, and the different exposures according to the geographic area. Specific climatic, edaphic, and hydrological factors also determine the incidence of leptospirosis in different habitats [34]. Some pathogens appear to have expanded their geographic or host range because of global warming and other associated climatic changes. Other contributing factors may include habitat changes caused by humans and resource depletion, causing the displacement of traditional wild hosts [35].

A green turtle that did not present anti-*Leptospira* antibody titers was sampled. In another survey, seropositivity to the bacteria was found in Blanding’s tortoises, in which all specimens were seropositive for *Leptospira* spp. and 73.5% of urine samples were positive to PCR [36]. In addition, in a study with red-eared sliders, 42 of 46 serum samples were seropositive for the serogroup Tarassovi [37].

In *Leptospira* infection, intraspecies and interspecies transmissions are dependent on the reservoir host animals, in which the bacteria can replicate and shed in urine over time, as well as the persistence of spirochetes in the environment and animal exposure [38]. Therefore, more research is needed to determine the role of wild animals in the epidemiology of leptospirosis [14]. According to a recent study in Chile, their prevalence is between 12.0% and 59.1%, between 3.0% and 25.2%, and between 23.3% and 65.4% in domestic animals such as dogs, domestic felines, and horses, respectively [39].

Overall, of the 15 seropositive wild animals, most had antibody titers for more than one serogroup because of two possible reasons: first, the animal reacted or was infected by more than one serogroup simultaneously. Second, it occurs because of cross-reactions [40], a phenomenon that is related to the acute phase of the disease, which can last from weeks to months, because as the disease becomes chronic, the antibodies that present the cross-reaction decrease. On the contrary, anti-*Leptospira* antibodies, which are more specific, tend to last for years in infected animals [41]. To determine with certainty the serogroup causing the infection, Chirathaworn et al. [40] recommended performing the isolation and molecular identification of the bacteria, but they highlighted the great usefulness of the MAT as a diagnostic method from an epidemiological point of view.

The most frequent serogroups detected were Tarassovi and Grippotyphosa. However, Tarassovi is considered the serogroup responsible for the reaction in 12 of the seropositive animals. These results are contrary to those of the systematic review of leptospirosis in wild animals in Latin America by Vieira et al. [14], who analyzed 79 articles published from 1975 to 2016 and studied four classes of animals, with mammals being the most reviewed, followed by amphibians, reptiles, and birds, where Icterohaemorrhagiae was described as the predominant serogroup. The serovars Tarassovi and Pomona have been related to pigs for decades [10]. Recently, Cilia et al. [25] confirmed that pigs are the maintenance hosts of Tarassovi and described it as being related to wild boars. In a report conducted in Slovenia, seroprevalence of 24.5% was obtained from a total of 437 wild boars for the serovar Tarassovi [42]. In Italy, seroprevalence of 3.14% from 39 wild boar serum samples was obtained for the serovar Tarassovi [34], and in an investigation in Vietnam, the serovar Tarassovi was found to be the most common in pigs, with a seropositivity of 2.19% in a total of 1959 serum samples [43]. Recently, Yupiana et al. [44] highlighted Tarassovi for its high prevalence and considered it an emerging serogroup of great importance in New Zealand cattle. In their study, in a total of 4000 dairy cows sampled, the prevalence of *Leptospira* spp. was 63.0% and seropositivity to Tarassovi was 17.0%.

A titer of 1:100 on the MAT is widely accepted as “positive”, and even lower titers can be considered exposures to *Leptospira* spp. because of the high specificity of the diagnostic test. Conversely, the antibody titers used to determine current or recent infection and titers used to establish that an animal has leptospirosis vary depending on the authors, including antibody titers from 1:200 to 1:1600 as indicators of possible infection [1,4,45,46]. According to the Leptospirosis Burden Epidemiology Reference Group, antibody titers ≥1:400 in endemic areas and ≥1:100 in nonendemic areas are considered cases of infection if they are accompanied by clinical signs [45]. For Levett [46], antibody titer values between 1:800 and 1:1600, in addition to the presentation of related clinical signs, are necessary to categorize an individual as having leptospirosis. Conversely, the World Organization for Animal Health [1] recognizes the MAT for diagnosing acute infection when a fourfold increase in antibody titers is demonstrated through paired serum samples. There is no consensus on the specific parameters used to determine exposure, infection, and disease in wildlife. Thus, in general, the antibody titers found in this research can be associated with infection by pathogenic *Leptospira* and even high titers of 1:800 and 1:1600 could provide evidence to determine leptospirosis in seropositive animals.

Regarding the demographic characteristics of the seropositive animals, a higher frequency of antibodies was present in adult animals (10/15), which could be related to the longer exposure time to the environment and maintenance hosts, in accordance with the findings of Fornazari et al. [47], although the role of age in the epidemiology of *Leptospira* spp. has not been sufficiently studied, especially in wild animals. Studies in rodents, didelphimorphs, and carnivorous mammals suggest a greater probability of exposure and development of chronic leptospirosis as the lifespan of the animals increases.

The causes of the entry of the birds to the wildlife rehabilitation center of the USS in the present study coincided with the findings of González-Acuña et al. [48], who analyzed the history of birds treated at the wildlife rehabilitation center of the University of Concepción, Chile, for 16 years, revealing that trauma was the most recurrent cause of admission, followed by orphanhood, with 58.2% and 22.7%, respectively. Conversely, the reason for the entry of the pudus seropositive for *Leptospira* is in accordance with the findings of Luarte and Leichtle [49] in the wildlife rehabilitation center of San Sebastián University located in the city of Concepción, Chile, where it was determined that dog attack was their main reason for admission. The reasons for the admission of the animals in the present study were not related to the presence of clinical signs of leptospirosis, but the finding of seropositivity is an indication of initial, active, or past infections, for which it would be important to establish the diagnosis of leptospirosis as a routine procedure in animals at the wildlife rehabilitation center. In a recent investigation conducted by Murillo et al. [50], acute-phase proteins [serum amyloid A (SAA), haptoglobin (Hp), albumin, and paraoxonase 1 (PON1)] were measured in domestic cats (*Felis catus*), postulating that they can aid in the diagnosis of leptospirosis because their levels can be used as an early marker for the disease and they can be used to assess inflammation, monitor the evolution of the infection, help in detecting subclinical infection, and differentiate acute from chronic diseases. It was found that their values were significantly higher in infected cats (recent or current infections). This finding could be useful for investigation in wild animals as a possible diagnostic test that would complement other methods such as the MAT or molecular techniques (PCR or bacteriological culture).

The seropositive animals were transferred from six communes that belong to the Los Lagos region in southern Chile to the wildlife rehabilitation center. This region and southern Chile, in general, have conditions that allow the dissemination of *Leptospira* spp. to the hosts and environment [9]. A study conducted in the Los Ríos region, wherein 104 water samples were collected in peridomiciliary environments, found pathogenic leptospires in water sources close to human populations [51]. The potential presence of reservoir wild animals and domestic animals, which are in constant contact, added to the abundance of wetlands and watercourses could influence in the probabilities of exposure or infection [9,52].

More research that includes the serial determination of leptospiral antibodies and/or the use of other diagnostic tests, such as bacteriological culture or molecular techniques, is recommended [1]. It is also suggested to conduct studies in pigs and wild boars to determine whether these species are the maintenance hosts for the serogroup Tarassovi [10,25,42,43] and to determine the role of these animals in the epidemiology of *Leptospira* spp.

Infectious diseases in wildlife populations can benefit efforts in conservation and provide a connection between serological studies and the well-recognized needs of detection, identification, and epidemiological surveillance [7]. Studies that include a larger sample size and other wildlife species to determine the prevalence of *Leptospira* spp. and the serogroups involved at the national level and studies focusing on the One Health approach are necessary. To achieve better control measures, coordination among animal and public health sectors, and an integrated policy, are needed [53].

## 5. Conclusions

Wild animals seropositive for pathogenic *Leptospira* are present in southern Chile. The overall seroprevalence was 25.00%. The seroprevalence values by class were 19.51% in birds, 38.89% in mammals, and 0% in reptiles. The most frequent serogroup detected was Tarassovi. The antibody titers in the seropositive animals were between 1:200 and 1:1600. Serological reactive animals showed different demographic characteristics.

Seropositivity was found in chilla foxes and pudus, and the serological reaction in a guiña was recorded for the first time. Seropositive birds of the species choroy parrots, bandurrias, and Magellanic penguins were also detected. These results highlight the exposure of different wild animal species to *Leptospira* infection, and it is important to continue this research to determine the consequences that infection may have from a clinical point of view in these species, as well as in the epidemiology of the disease due to possible intra- and interspecies transmission, its zoonotic potential and public health relevance at the local level and worldwide, as well as the possible role of wild animals as sentinels for *Leptospira* infection.

## Figures and Tables

**Table 1:** Seroprevalence of pathogenic *Leptospira* of the total sampled animals and specific seroprevalence by animal class, with their corresponding 95% CI. Wildlife rehabilitation center, USS, campus Patagonia, Puerto Montt, Chile. Data range: January to May 2023.

Class	Seropositive Animals	Sampled Animals	Seroprevalence	95% CI
Birds	8	41	19.51	7.37–31.64
Mammals	7	18	38.89 *	16.37–61.40
Reptiles	0	1	0	…
Total	15	60	25.00	14.04–35.96

* *p* value > 0.05.

**Table 2:** Species that were part of the study, number of animals seropositive for pathogenic *Leptospira*, and total number of animals sampled by species, with their corresponding seroprevalence and 95% confidence intervals (95% CI). Wildlife rehabilitation center, USS, campus Patagonia, Puerto Montt, Chile. Data range: January to May 2023.

Species	Number of Seropositive Animals	Number of Sampled Animals	Seroprevalence (%)	95% CI
Choroy parrot	3	9	33.33	2.53–64.13
Bandurria	3	13	23.08	0.18–45.98
Magellanic penguin	2	3	66.67 *	13.33–100
Owl	0	2	0	…
Dominican seagull	0	2	0	…
Huairavo	0	1	0	…
Tiuque	0	6	0	…
Peregrine falcon	0	1	0	…
Blanquillo	0	1	0	…
Chilean salter	0	1	0	…
Traro	0	1	0	…
Black petrel	0	1	0	…
Chilla fox	4	9	44.44	11.98–76.90
Pudu	2	5	40.00	0.00–82.94
Guiña	1	2	50.00 *	0.0–100
South American sea lion	0	1	0	…
Andean fox	0	1	0	…
Green turtle	0	1	0	…

* *p* value > 0.05.

**Table 3:** Summary of the seropositive animals for pathogenic *Leptospira* with the highest titer for a determined serogroup. Wildlife rehabilitation center, USS, campus Patagonia, Puerto Montt, Chile. Data range: January to May 2023.

Species	Serogroup and Antibody Titer (MAT)
Choroy parrot 1	Tarassovi = 1:800
Choroy parrot 2	Tarassovi = 1:400
Choroy parrot 3	Tarassovi = 1:400
Bandurria 1	Tarassovi = 1:400
Bandurria 2	Grippotyphosa = 1:100
Bandurria 3	Tarassovi = 1:200
Magellanic penguin 1	Tarassovi = 1:1600
Magellanic penguin 2	Tarassovi = 1:200
Chilla fox 1	Tarassovi/Sejroe = 1:400
Chilla fox 2	Sejroe = 1:800
Chilla fox 3	Tarassovi = 1:400
Chilla fox 4	Tarassovi = 1:400
Pudu 1	Tarassovi = 1:800
Pudu 2	Tarassovi = 1:400
Guiña	Tarassovi = 1:800

## Data Availability

The data presented in this study are publicly available.
